# Comparative Analysis of Clinical and Imaging Features of Osteomalacia and Spondyloarthritis

**DOI:** 10.3389/fmed.2021.680598

**Published:** 2021-05-20

**Authors:** Zheng Zhao, Wenji Chen, Yanyan Wang, Jingyu Jin, Yurong Zhao, Jian Zhu, Feng Huang

**Affiliations:** ^1^Department of Rheumatology and Immunology, The First Medical Center of PLA General Hospital, Beijing, China; ^2^Department of Rheumatology and Immunology, Hainan Hospital of PLA General Hospital, Hainan, China

**Keywords:** bone mineral density, comparative analysis, osteomalacia, sacroiliac-joint magnetic-resonance imaging, spondyloarthritis

## Abstract

**Aim:** To compare the clinical and radiological characteristics of osteomalacia and spondyloarthritis/ankylosing spondylitis (SpA/AS) in order to provide a basis for differential diagnosis.

**Methods:** We carried out a retrospective analysis of patients who were diagnosed with osteomalacia at the First Medical Center of 301 Hospital (Beijing, China) from January 2012 to January 2019. The clinical and radiological data of all patients were collected; at the same time, we selected age- and gender-matched patients with SpA/AS for comparison.

**Results:** We enrolled a total of 76 patients, 38 with osteomalacia, and 38 with SpA/AS. The mean ages of the two groups were, respectively 44.62 ± 14.90 years and 44.85 ± 9.76 years (*P* > 0.05). Of patients with osteomalacia, 65.79% (*n* = 25) had previously been misdiagnosed with SpA/AS. In the osteomalacia and SpA/AS groups, there were, respectively 31 and 33 patients with low back pain, 22 and 13 patients with peripheral arthralgia, and 13 and 3 patients with heel pain. Alkaline phosphatase (ALP) level was significantly higher in the osteomalacia than in the SpA/AS group (*P* < 0.05). Serum phosphorus levels, erythrocyte sedimentation rate (ESR), C-reactive protein (CRP) levels, and bone mineral density (BMD) were significantly lower in the osteomalacia group than the SpA/AS group (*P* < 0.05). Twenty-five patients in the osteomalacia group underwent sacroiliac-joint magnetic-resonance imaging (SIJ–MRI); abnormalities were found in 10 of these patients, seven of whom met the definition for positive SIJ–MRI according to 2009 Assessment of SpondyloArthritis international Society (ASAS) criteria. All seven presented with bilateral sacral involvement. Logistic-regression analysis found that the odds ratio (OR) for bone erosion score was 0.551; the higher this score, the lower the possibility of osteomalacia.

**Conclusion:** Clinical and radiological presentations of patients with osteomalacia could highly simulate those of patients with spondyloarthritis; identifying the differences between these two diseases could effectively decrease the misdiagnosis rate.

## Introduction

Bone is hard tissue formed by hydroxyapatite crystals [Ca_10_(PO_4_)_6_(OH)_2_] deposited on matrix protein produced by osteoblasts. There is an increase in the unmineralized bone matrix in rickets, and osteomalacia is developed due to the damage of matrix protein mineralization (1, 2). Among them, rickets occurs in children, and osteomalacia is a disease in adulthood. Osteomalacia is a rare disease. Patients may complain of bone pain, myasthenia, arthralgia, and/or scoliosis. The clinical and even radiological presentations of osteomalacia can simulate those of spondyloarthritis, but the two diseases are different. Patients with early-stage osteomalacia can recover completely, while untreated patients can become completely bedridden due to severe bone pain. However, there are still no unified international criteria for the diagnosis of osteomalacia, resulting in misdiagnosis and therefore delayed treatment of some patients. In this paper, we summarize patients with osteomalacia treated at our hospital over the last 7 years. Our results showed that 65.79% of them had been previously misdiagnosed with spondyloarthritis/ankylosing spondylitis (SpA/AS). Therefore, herein we compare the clinical, laboratory, and radiological characteristics of this group of patients with those of matched patients with SpA/AS, in order to decrease misdiagnosis in patients with osteomalacia and improve their outcomes.

## Subjects and Methods

### Patient Selection

Patients diagnosed with osteomalacia at the First Medical Center of 301 Hospital (Beijing, China) from January 2012 to January 2019 were included in this study. The diagnosis of osteomalacia is defined as (3): (1) hypophosphatemia or hypocalcemia; (2) high ALP; (3) clinical presentation of myasthenia or bone pain; (4) BMD <80% of the normal level for the patient's age group; (5) bone scan showing multiple metabolic abnormalities or X-ray showing multiple osteoporosis sites. A definitive diagnosis of osteomalacia can be obtained if criteria 1–5 are met. Subjects without clinical symptoms, low BMD, and radiological abnormalities (3–5) might have osteomalacia. At the same time, we also selected age- and gender-matched patients with SpA/AS, and all patients with SpA met the 2009 ASAS classification criteria for axial spondyloarthritis.

### Methods

We obtained and recorded data on patients' genders, ages, clinical-presentation data, biochemical markers [blood phosphorus, blood calcium, serum creatinine (sCr), and serum alkaline phosphatase (ALP)], bone mineral density (BMD), and sacroiliac-joint magnetic-resonance imaging (SIJ–MRI) results. BMD was represented by T score in L1-L4 spine. The Spondyloarthritis Research Consortium of Canada (SPARCC) scoring system was used to score SIJ–MRI results. The current study was carried out based on the approval of the Ethics Committee of The first Medical Center of PLA General Hospital (Beijing, China). All participants provided written informed consent prior to the start of this study.

### Statistical Analysis

We used SPSS version 19.0 (IBM Corp., Armonk, NY, USA) for statistical processing of data. Quantitative data were expressed as mean ± standard deviation (SD), while qualitative data were described using frequency (percentage). Student's *t*-test or a rank-sum test was used for statistical analysis. We used logistic-regression analysis to identify risk markers for osteomalacia.

## Results

### General Information

A total of 38 patients with osteomalacia were included; 17 cases were due to adefovir use, 13 were due to tumors, and eight had unknown etiologies. Simultaneously, we selected 38 age- and gender-matched patients with SpA/AS for comparison (Table 1). Results showed no significant difference in mean age or gender between the two groups. However, the mean disease duration of patients with osteomalacia was shorter than that of patients with SpA/AS.

**Table 1:** General information of patients in both groups.

	**Osteomalacia**	**SpA/AS**	***P***
Age	44.62 ± 14.90	44.85 ± 9.76	0.934
Gender (male/female)	25/13	29/9	0.312
Disease duration (months)	34.37 ± 47.12	195.45 ± 116.88	0.000

### Comparison of Clinical Symptoms Between the Two Groups

The main clinical symptoms of patients with osteomalacia and SpA/AS were lumbago, peripheral arthralgia, and hip pain. Lumbago and hip pain had no difference between patients with osteomalacia and SpA/AS. More patients with osteomalacia developed peripheral arthralgia than those with SpA/AS. Heel pain and fractures were significantly uncommon in patients with SpA/AS than in those with osteomalacia. In addition, patients with osteomalacia but not those with SpA/AS experienced generalized pain and myasthenia (Table 2).

**Table 2:** Clinical symptoms of patients in both groups.

	**Osteomalacia (*n*)**	**SpA/AS (*n*)**	***P***
Lumbago	31	33	0.529
Peripheral arthralgia	22	13	0.038
Hip pain	3	5	0.455
Heel pain	13	3	0.028
Fractures	9	1	0.007
Generalized pain	8	0	–
Myasthenia	6	0	–

### Comparison of Blood Test Results Between the Two Groups

Serum ALP was significantly higher in patients with osteomalacia than in those with SpA/AS (*P* < 0.01). Serum phosphorus levels, erythrocyte sedimentation rate (ESR), C-reactive protein (CRP) levels, human leukocyte antigen (HLA)–B27 positivity rate, and BMD of the lumbar vertebrae and femoral neck were significantly lower in osteomalacia than in patients with SpA/AS (*P* < 0.05; Table 3).

**Table 3:** Laboratory test results of patients in both groups.

	**Osteomalacia**	**SpA/AS**	***P***
AKP (μmol/L)	418.47 ± 326.25	86.72 ± 45.43	0.000
Serum phosphorus	0.47 ± 0.14	1.29 ± 0.6	0.000
Serum calcium	2.18 ± 0.20	2.27 ± 0.09	0.248
Creatinine	82.52 ± 33.88	73.89 ± 16.00	0.151
25-Hydroxyvitamin D3 (ng/ml)	17.47 ± 10.04	21.83 ± 6.04	0.295
Erythrocyte sedimentation rate	5.50 ± 6.19	21.66 ± 20.22	0.002
CRP	0.74 ± 0.02	2.26 ± 2.58	0.016
HLA-B27 (*n*)	0.03% (1)	97.37% (37)	0.000
Lumbar vertebral bone mineral density	−3.23 ± 1.44	−1.81 ± 0.92	0.000
Femoral neck bone mineral density	−3.45 ± 0.91	−1.38 ± 0.63	0.000

### Comparison of SIJ–MRI Results of the Two Groups

#### Comparison of SIJ–MRI Abnormalities Between the Two Groups

From the osteomalacia and SpA/AS groups, respectively 25 and 33 patients underwent SIJ–MRI examination; 40% (*n* = 10) and 54.5% (*n* = 18) patients were, respectively found to have abnormal presentations. There was no significant difference between the two groups (*P* = 0.272).

#### Comparison of SIJ–MRI Scores Between the Two Groups

**Figure 1:**
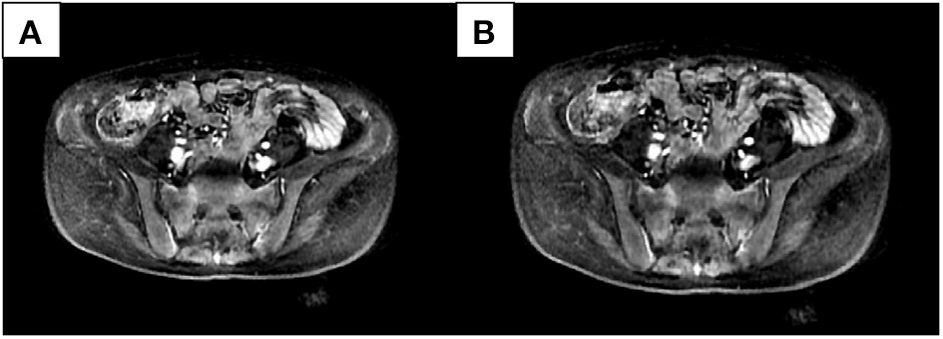
Female, 28 years old, chief complaint of lumbago for 21 months, exacerbation accompanied by generalized asthenia for 6 months. Erythrocyte sedimentation rate, C-reactive protein, and human leukocyte antigen-B27 were all negative. Alkaline phosphatase was 269.6 U/L; inorganic phosphorus was 0.43 mmol/L. Bilateral sacral bone marrow edema signals could be seen in two continuous layers **(A,B)** in the short-tau inversion recovery sequence.

We compared patients with SIJ–MRI abnormalities between the two groups (10 in the osteomalacia group, and 18 in the SpA/AS group). In the osteomalacia group, seven patients met the definition for positive SIJ–MRI according to 2009 ASAS criteria (Figure 1), showing bilateral sacral involvement, and three (3/7) showed bone erosion. There was no significant difference in bone marrow edema (BME) signal score in SIJ–MRI between the two groups. However, bone erosion score was lower in the osteomalacia than the SpA/AS group (*P* < 0.05; Table 4).

**Table 4:** SIJ–MRI scores of patients in both groups.

	**Osteomalacia**	**SpA/AS**
BME	8.20 ± 7.80	5.67 ± 12.46
EROSION	1.50 ± 1.71	4.29 ± 3.99^*^

* *P < 0.05 when the two groups were compared*.

#### Comparison of SIJ Involvement Between the Two Groups

The involvement site in patients with osteomalacia was the bilateral sacral bone; iliac involvement was absent in all 10 patients. Table 5 shows the involvement statuses of the two groups. Both tended to have bilateral SIJ involvement, but abnormal changes in the osteomalacia group tended to be focused on the sacrum, while sacral and iliac involvement were present in patients with SpA/AS. There was a significant difference between the two groups.

**Table 5:** Distribution of SIJ involvement in patients in both groups.

	**Osteomalacia (*****n*****)**		**SpA/AS (*****n*****)**	
	**Bilateral**	**Unilateral**	**Bilateral**	**Unilateral**
	10	0	16	2
Sacral involvement	10^*^	0	1	1
Iliac involvement	0	0	2	0
Sacral/iliac involvement	0	0	13^*^	1

* *P < 0.01 when the two groups were compared*.

### Predictive Significance of SIJ–MRI for Osteomalacia

**Figure 2:**
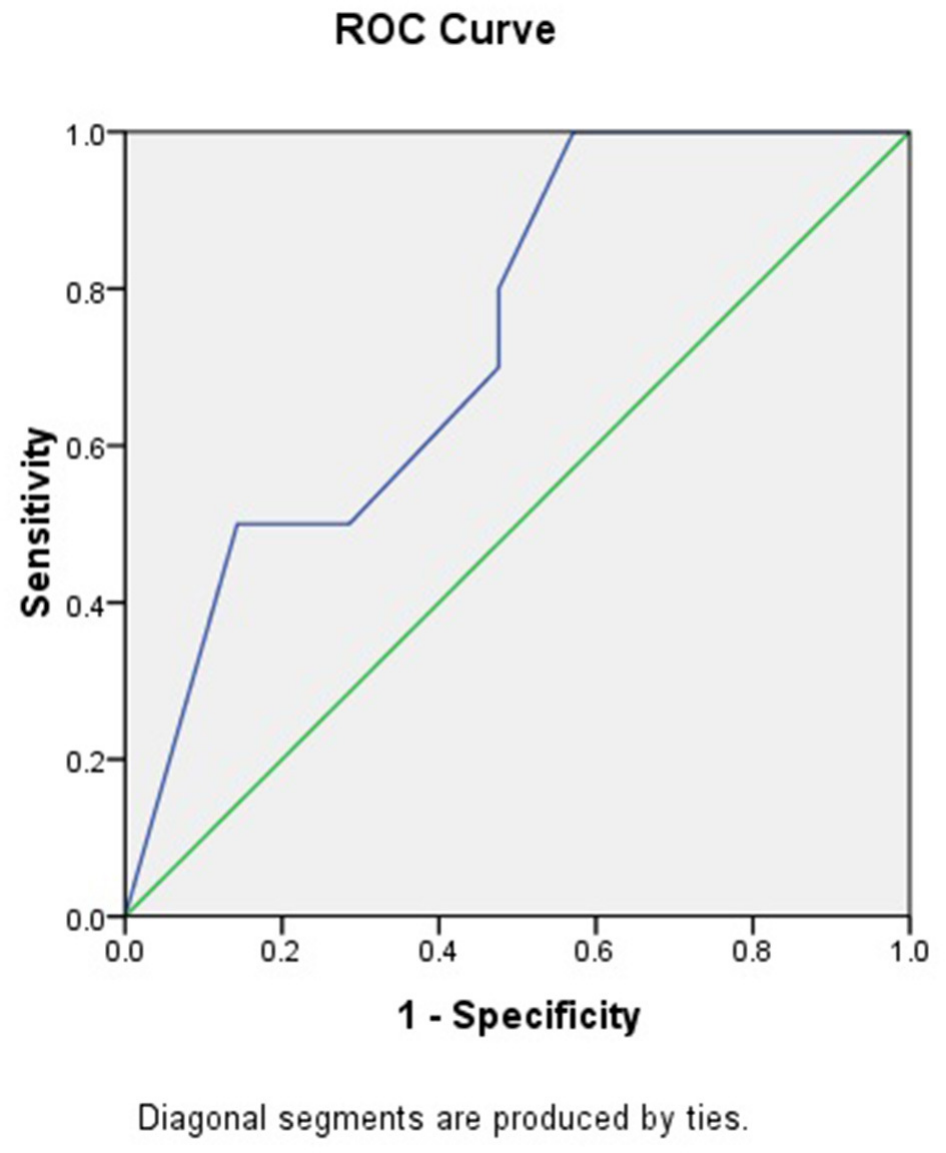
ROC curve of SIJ–MRI bone erosion scores.

Ten patients from the osteomalacia group and 18 from the SpA/AS group had abnormal SIJ–MRI presentations. We performed logistic-regression analysis of SIJ–MRI abnormalities (i.e., BME and bone erosion) in both groups. ORs were, respectively 0.995 (CI: 0.939–1.088) and 0.551 (CI: 0.3250–0.934), with respective *P*-values of 0.921 and 0.027. In other words, the higher the bone erosion scores, the lower the possibility of osteomalacia. We performed receiver-operating characteristic (ROC) curve analysis on bone erosion scores and obtained an area under the curve (AUC) of 0.736 (CI: 0.557–0.914; Figure 2). When bone erosion score was 3.5, sensitivity and specificity of osteomalacia diagnosis were 80.0 and 61.9%, respectively.

## Discussion

Osteomalacia is classified as a metabolic bone disorder, but endocrinologists do not consider it an endocrine disorder. History demonstrates that vitamin D deficiency causes rickets and osteomalacia, a fact that is extremely important in clinical practice (4). Studies on vitamin D deficiency led to the identification of 25-hydroxyvitamin D [25(OH)D] and 1, 25-dihydroxy vitamin D [1, 25(OH)2D]. The etiologies of osteomalacia were subsequently discovered, including drug-induced and tumor-induced osteomalacia. In our study, we included 17 patients with drug (adefovir)–induced osteomalacia and 13 with tumor-induced osteomalacia. Thyroid and parathyroid function tests were performed for all patients after osteomalacia was considered, but no significant abnormalities were found. Therefore, we could not exclude tumor-induced osteomalacia in the eight patients with unknown etiologies. The distribution of such tumors is not specific, and the tumors themselves are not easily found. The most common presentations of osteomalacia are chronic lumbago, peripheral arthralgia, generalized pain, and myasthenia. These presentations are non-specific, which is why this disease tends to be misdiagnosed.

In the early stage of SpA/AS, there are only chronic low back pain, mainly involving sacroiliac joint and axial bone, as well as peripheral joint involvement, which is difficult to distinguish from osteomalacia in clinical manifestation. We included 38 patients with osteomalacia in this study with a mean age of 44.62 ± 14.90 years. One patient was HLA-B27^+^, 31 patients experienced lumbago, 22 experienced peripheral arthralgia, and 13 experienced heel pain. These symptoms are highly similar to those of SpA/AS, which is why 65.79% (25/38) of our patients with osteomalacia had been misdiagnosed with SpA/AS. In order to compare clinical and radiological differences between the two diseases, we selected patients with SpA/AS matched by gender and age with the osteomalacia group. Results showed that the disease duration of these patients was significantly longer than that of patients with osteomalacia. This could be because SpA/AS mostly occurs in young adults, age 20–30 years, while the mean age of our selected patients was 44.85 ± 9.7 years. Blood test abnormalities, such as serum ALP, phosphorus, ESR, and CRP levels, in patients with SpA/AS with long disease duration can differ from those of early-stage patients, as the former can develop osteoporosis. Studies show that the incidences of osteoporosis in SpA/AS are 11.7–34.4% (5). Our study also found decreased BMD in patients with SpA/AS. However, BMD reduction was more significant in patients with osteomalacia (Table 3).

**Figure 3:**
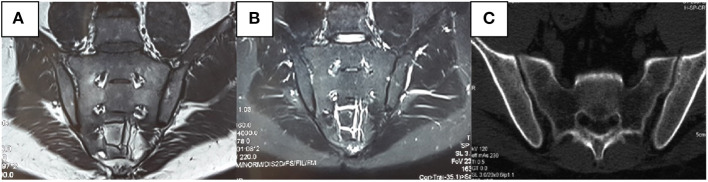
Male, 31 years old, bilateral hip pain for 8 months and lumbago for 2 months. C-reactive protein, rheumatoid factors, and human leukocyte antigen-B27 were all negative. Alkaline phosphatase was 346 U/L. Inorganic phosphorus was 0.48 mmol/L. No apparent abnormalities were observed in the sacroiliac joint (SIJ)–magnetic resonance imaging T1-weighted [T1W, **(A)**] and short-tau inversion recovery [STIR, **(B)**] sequences. It can be seen in the computerized tomography that the left SIJ is coarse rather than smooth **(C)**.

We also found that radiological abnormalities were another main cause of misdiagnosis, in addition to clinical symptoms. Five patients in our study had been misdiagnosed with SpA/AS due to SIJ–computed-tomography (CT)/X-ray abnormalities. Abnormalities in conventional radiology mainly present as coarse SIJ with a lack of smooth bone (Figure 3). Ten patients had been misdiagnosed due to SIJ–MRI abnormalities, seven of whom met the definition for positive SIJ–MRI for patients with SpA (6). In 2019, the ASAS–MRI working group redefined SIJ–MRI abnormalities (7). The group believed that even though SIJ–MRI presentation might suggest SpA, there is a need to combine clinical presentation, laboratory tests, and other markers. Therefore, osteoarthritis, infection, trauma, tumors, and artifacts in osteomalacia can mimic MRI presentations of patients with SpA. In our current study, we found that patients with osteomalacia could develop BME, but the OR of this finding was 0.995 (CI: 0.939–1.088). Therefore, BME on MRI has little risk for osteomalacia. However, bone erosion score in osteomalacia was significantly lower than in SpA/AS; its OR was 0.551 (CI: 0.325–0.934), and the area under the ROC curve was 0.736 (CI: 0.557–0.914). Therefore, the higher the bone erosion score, the lower the risk of osteomalacia, but sensitivity and specificity of this finding were not high. The longer disease duration of patients with SpA/AS enrolled in our study might be a reason for the higher bone erosion score. Moreover, erosion itself in sacroiliac joints can occur in many clinical situations and observed in osteomalacia as a decrease of BMD. Therefore, this marker might not be suitable for differentiating between osteomalacia and early-stage patients with SpA. It is worth noting that fatty infiltration occurred in only one patients in the osteomalacia group, and joint fusion did not occur in in this group at all. As fatty infiltration is not a specific radiological presentation in patients with SpA/AS, while SIJ fusion is one of the most specific presentations of advanced AS, and as our matched patients with SpA/AS had a mean disease duration of ≥16 years, many of these patients suffered from joint fusion. In order to exclude this effect, we did not include those two scores in this study. We also found that MRI abnormalities in patients with osteomalacia were mainly concentrated on the sacrum, and bilaterally symmetrical involvement was present. In contrast, although patients with SpA/AS also showed bilateral involvement, both sacral involvement and iliac involvement were sometimes present. This was the greatest difference in SIJ–MRI findings between the two groups (Table 5). Therefore, we speculate that the site of SIJ–MRI abnormalities could have greater significance in osteomalacia diagnosis than in SpA/AS diagnosis. We also found that SIJ–MRI abnormalities mostly occurred in tumor-induced osteomalacia and were rare in drug-induced osteomalacia. A recent study showed that fibroblast growth factor 23 (FGF23) is a phosphatonin produced by bones, the excessive effects of which cause many types of hypophosphatemic rickets and osteomalacia (8). This could be the pathological basis for tumor-induced osteomalacia and also a reason for the greater tendency toward radiological abnormalities associated with this etiology. Moreover, the errors in the interpretation of both radiologists and rheumatologists on the images should also not be overlooked, and their errors occurred while there was not enough experience in the SIJ–MRI.

At present, no single laboratory test can demonstrate the presence of osteomalacia. However, patients with osteomalacia often present with high ALP and either hypophosphatemia or hypocalcemia; we saw similar results in this study (Table 3). In addition, we found no significant difference in 25-hydroxyvitamin D3 levels between the two groups, but ESR and CRP were significantly lower in the osteomalacia than in the SpA/AS group (Table 3). This showed that osteomalacia was a non-inflammatory lesion. It is currently believed that hypophosphatemia or hypocalcemia and high ALP are essential to osteomalacia diagnosis or suspicion thereof. However, their significance in such diagnosis, particularly differential diagnosis, is still not widely recognized. At present, there is no unified diagnostic standard of osteomalacia in the world. A Japanese study defined the diagnosis of osteomalacia (3), and we also adopted this diagnostic criteria as the inclusion criteria, but this study also pointed out that osteomalacia should be distinguished from SpA/AS in addition to other endocrine diseases.

This study have some strengths and limitations. As a strength, highlighted the importance of differential diagnosis between these conditions due to the overlap of clinical manifestations between osteomalacia and SpA/AS. However, all retrospective clinical works, including this study, are subject to data collection failures. Moreover, there is no comment about image data on presence or absence of syndesmophytes and spine examination in this study. Finally, this study did not take into account different therapeutics and SpA disease activity index. Long term disease and low disease activity, secondary to therapeutic methods or controlled disease, may be related to less decreased gap of SIJ BME between groups.

## Conclusions

Osteomalacia is an uncommon disease but can severely affect patients' quality of life. Untreated patients with osteomalacia can become completely bedridden due to severe myasthenia and bone pain. However, those in the early stage can make a full recovery. A lack of specific clinical, laboratory, and radiological abnormalities, as well as of unified diagnostic criteria, pose a challenge in early and accurate diagnosis for clinicians. Our study showed that the clinical presentations of patients with osteomalacia had many similarities with those of SpA/AS, but only patients with osteomalacia developed generalized pain and myasthenia. At the same time, inflammatory markers were not elevated in patients with osteomalacia. Radiological abnormalities, particularly SIJ–MRI abnormalities, were one of the main causes of osteomalacia being misdiagnosed as SpA/AS. The SIJ–MRI presentation of osteomalacia might meet the definition for positive MRI in patients with SpA/AS proposed by the ASAS–MRI working group, but lesions in patients with osteomalacia mainly occur on the sacrum. Low levels of serum phosphorus and serum calcium and high levels of ALP can help physicians effectively distinguish these two diseases. Therefore, we recommend that all patients with lumbago or suspected SpA/AS undergo serum phosphorus, serum calcium, and ALP tests. These simple serological tests could effectively decrease the risk of osteomalacia misdiagnosis.

## Data Availability Statement

The original contributions generated for the study are included in the article/supplementary material, further inquiries can be directed to the corresponding author/s.

## Ethics Statement

The studies involving human participants were reviewed and approved by the First Medical Center of PLA General Hospital. The patients/participants provided their written informed consent to participate in this study.

## Author Contributions

ZZ, WC, YW, JJ, YZ, JZ, and FH contributed to the study design, conducted the data collection and analyses, and drafted the paper. All authors contributed to the article and approved the submitted version.

## Conflict of Interest

The authors declare that the research was conducted in the absence of any commercial or financial relationships that could be construed as a potential conflict of interest.
